# The role of orthodontists in the multidisciplinary management of obstructive sleep apnea

**DOI:** 10.1186/s40510-024-00541-3

**Published:** 2024-11-04

**Authors:** Jorge Faber, Aliciane Mota, Lai-In Ho, M Ali Darendeliler

**Affiliations:** 1 University of Brasília, Brasília, Brazil; 2 Hong Kong Children’s Hospital, Hong Kong, Hong Kong; 3 The University of Sydney, Sydney, Australia

**Keywords:** Orthodontics, Obstructive sleep apnea, Multidisciplinary management, Maxillomandibular advancement, Mandibular advancement appliances, Rapid maxillary expansion

## Abstract

**Background:**

Obstructive sleep apnea (OSA) is a complex disorder characterized by interruptions in breathing during sleep, leading to a range of adverse outcomes from reduced quality of life to serious health risks, including cardiovascular diseases and increased mortality.

**Main Body:**

This manuscript reviews the orthodontists’ essential role in the multidisciplinary healthcare team tasked with managing OSA. It particularly highlights critical orthodontic interventions, such as surgical-orthodontic maxillomandibular advancement (MMA), mandibular advancement appliances (MAAs), and rapid maxillary expansion (RME). These interventions are pivotal in modifying craniofacial structures to enhance airway patency. The importance of conducting a thorough airway analysis is underscored, assessing the complete anatomical and functional factors contributing to airway obstruction.

**Conclusion:**

The paper calls for increased collaborative research efforts to develop standardized, evidence-based orthodontic procedures for effectively managing OSA, aiming to improve patient outcomes through specialized, tailored interventions.

## Introduction

Obstructive sleep apnea (OSA) has a broad spectrum of consequences. On the milder end, this disorder can impair the daily quality of life for affected individuals, primarily manifesting as excessive daytime sleepiness. In more severe cases, however, it can predispose patients to a variety of health complications [1–3] that, in the most critical scenarios, may lead to fatalities [4, 5]. This range of impacts underscores the importance of understanding, diagnosing, and appropriately treating sleep apnea, given its significant potential to affect morbidity and mortality.

One of the most used variables in the analysis of sleep apnea severity is the Apnea-Hypopnea Index (AHI). The AHI is a metric used to assess the severity of sleep apnea by calculating the average number of apnea and hypopnea events per hour of sleep. In adults, the severity is classified as normal (AHI < 5), mild (5 ≤ AHI < 15), moderate (15 ≤ AHI < 30), or severe (AHI ≥ 30).

OSA arises from increased collapsibility of the upper airway [6], which serves as a metric for the structural burden of the upper airway [7]. No single anatomical variable strongly predicts upper airway (UA) collapsibility alone [8], yet there are numerous predictors of increased UA collapsibility. For instance, tongue size and tongue fat [9] have been correlated with the Apnea-Hypopnea Index (AHI), as well as pterygomandibular fat [10]. Tongue fat contributes to a larger tongue size and might reduce tongue force, impairing its ability to function effectively as an upper airway dilator. Enlarged tonsils were associated with OSA in both children [11] and adults [12]; Craniofacial skeletal morphology has been demonstrated to play an important role in the patency of the upper airway in both children and adults, being decreased mandibular and maxillary lengths and increased face height associated with OSA [13], as well as the reduced sagittal diameter of the oropharynx, avoid airway collapse increased gonial angle, and hyoid position [14].

An additional endotype observed in OSA patients features a narrow maxilla [15–17] accompanied by a high-arched palate. These conditions correlate with elevated nasal resistance and the displacement of the tongue towards the posterior. These specific anatomical characteristics are associated with OSA across various age groups [6]. 

It comes as no surprise that the essential team for managing this condition is comprised of a diverse group of specialists: endocrinologists, psychiatrists, psychologists, nutritionists, myofunctional therapists, neurologists, otolaryngologists, pulmonologists, and dentists, often including orthodontists but not limited to them. The treatment of OSA stands out as a condition that necessitates a uniquely multidisciplinary approach, highlighting the critical need for a seamlessly integrated collaborative effort among various healthcare professionals to tackle the complexity of this disorder effectively.

Building on these principles, this article aims to provide an overview of an orthodontist’s roles within the multidisciplinary team treating OSA. It is important to note that while Continuous Positive Airway Pressure (CPAP) therapy is an excellent treatment option for OSA, it falls outside the orthodontist’s scope of practice and will not be discussed in this context. Despite its applicability to many patients, the discussion will also exclude weight loss and sleep posture recommendations, even though lateral positioning can avoid airway collapse. Professional guidance on weight loss and adjustments in sleeping posture should always be considered in patient recommendations when appropriate, highlighting the need for a comprehensive approach to patient care within the multidisciplinary framework.

This article aims to explore the most common surgical and non-surgical treatments employed by orthodontists in managing obstructive sleep apnea (OSA). While Continuous Positive Airway Pressure (CPAP) therapy is a well-established treatment for OSA, it lies outside the scope of orthodontic practice and will not be addressed. Similarly, weight management and sleep posture modifications are crucial in the overall treatment of OSA. Still, they will not be covered here, even though orthodontists can guide patients regarding weight loss and the importance of sleep posture adjustments. Instead, this article will focus specifically on orthodontic interventions. By examining both well-established and emerging techniques, it seeks to provide a comprehensive overview of the orthodontist’s role within the multidisciplinary team involved in OSA management.

## Surgical-orthodontic maxillomandibular advancement

Surgical maxillomandibular advancement (MMA) is one of OSA’s most effective anatomic surgical interventions. In the early 1980s, several studies highlighted improvements in polysomnographic parameters among patients undergoing mandibular osteotomy with advancement [18–20]. Nonetheless, by the mid-1980s, combined maxillomandibular advancement gained preference over solely mandibular osteotomy for treating nonsyndromic OSA patients [21, 22]. This shift was motivated by the desire to maintain the maxillary-mandibular relationship and the growing understanding that the physiological etiology of OSA frequently involves both mandibular and maxillary deficiencies.

tongutonguePrecise phenotyping of patients with polysomnographic findings, targeted physical examination, and diagnostic tools are critical to optimizing surgical success in patients with OSA [23]. MMA redesigns the airway, increasing its volume and/or reducing collapsibility. While this therapeutic approach is most often employed in the presence of skeletal disharmonies, it can sometimes be applied even without such disharmony. This recommendation typically arises when a patient is intolerant of or unable to use CPAP or a mandibular advancement appliance.

The skeletal changes resulting from MMA typically modify the dental relationship, necessitating occlusal correction through orthodontic treatment [24]. Due to the urgency of treating the apnea, the management of OSA may require a deviation from traditional orthodontic approaches.

Three main strategies are identified in the context of integrating orthodontic treatment with surgical interventions for OSA patients. The first involves conducting surgery without any prior orthodontic planning. The second approach, termed the “Surgery First” protocol, anticipates that most, if not all, orthodontic dental movements will be performed after surgery. This strategy requires extensive orthodontic treatment planning before the surgical procedure, with orthodontic appliances being fitted before the surgery to ensure the start of the orthodontic treatment just after surgery [25]. The third strategy includes traditional orthodontic preparation before undertaking skeletal correction surgery. This approach, however, should be reserved for cases where the patient can use a CPAP throughout the entire orthodontic preparatory period.

There is a notable gap in research directly comparing the various orthodontic strategies for the surgical-orthodontic management of OSA. As a result, selecting an orthodontic treatment approach frequently depends on the clinician’s expertise and capability to execute one of these methods. Additionally, patient preferences play a crucial role, especially when making informed decisions about their treatment options. This emphasizes the importance of a patient-centered approach in choosing the most suitable treatment plan.

## Mandibular advancement appliances

Not all patients are able or willing to undergo surgery. In this context, Mandibular Advancement Appliances (MAAs) have emerged as a treatment option for adults, with the first clinical studies appearing in the 1990s [26, 27]. These devices maintain the jaw in a forward position during sleep and are based on the design of functional appliances used to treat Class II malocclusions in growing patients [28, 29]. To a lesser extent, these devices mimic the mandibular movement that would occur if the patient were to undergo mandibular advancement surgery [20]. Imaging of the airway has shown that anterior mandibular protrusion primarily increases the airway’s caliber in the retropalatal area through lateral expansion and the displacement of parapharyngeal fat pads, while the tongue and base-of-tongue muscles also move forward [30, 31]. 

Treatment with MAA begins with obtaining a bite registration with the mandible advanced, typically set at about 50–75% of the maximum advancement potential [32, 33]. Following the appliance’s fitting at this initial advancement level, incremental advancements are made to increase the mandibular projection gradually. This stepwise process is designed to allow the patient to adapt to the device, increasing joint mobility and enabling the use of the appliance at greater advancement levels. The term “titration” refers to this systematic process of making incremental advancements until a degree of advancement is achieved that successfully reduces obstructive events. This acclimatization period aims to find the mandible’s most efficient and comfortable position. The titration process typically spans from 3 to 6 months [34]. 

Once the patient reaches a level of advancement where they report subjective improvements in sleep quality and a reduction in snoring, it is advisable to conduct a follow-up polysomnography. This is to assess the necessity for further advancements or to determine if the current position can be established as the optimal treatment setting. This approach ensures a tailored treatment plan that not only aims to effectively reduce OSA symptoms but also maximizes patient comfort and compliance.

The MAA is typically used continuously over extended periods and represents a robust treatment modality for OSA [35–38]. The success of the treatment is achieved by reducing OSA severity when the patient uses the device, similar to how glasses only improve vision while being worn. In other words, the device is not intended to cure the patient with sleep apnea so that they could one day be free of the disorder and no longer require the use of the appliance.

MAAs are primarily recommended for patients with primary snoring and mild to moderate apnea. However, when MAAs are used in cases of severe apnea where individuals are intolerant or non-responsive to CPAP, they tend to offer significant benefits [4]. Mehta et al., in their 2001 study, found that MAA was more efficient in severe OSA cases than in mild OSA cases [32]. High air pressure is one of the common complaints among individuals who are intolerant of CPAP. Combination therapy of MAA and PAP would reduce the optimal airway pressure [39]. This combination therapy should be considered for patients with severe OSA who have incomplete responses to MAA therapy alone and are intolerant of PAP therapy.

MAAs are highly tolerable and have relatively few joint repercussions [40], but a common side effect is dental movement. After ten years of MAA use, a mean decrease in overjet by 1.5 mm has been reported [41]. Should dental changes occur, the patient should consult their dentist to decide whether to continue using the device or discontinue it in favor of switching to another therapy. It is worth noting that several patients, advised by their dentists, opt to continue using the device due to the significant improvements in the quality of life it provides.

Recent scientific publications have introduced mandibular advancement appliances in growing patients, often in combination with rapid maxillary expansion procedures [42–44]. However, doubts remain whether the long-term outcomes of these devices exceed those achievable through maxillary expansion alone. This skepticism arises partly because, while MAAs can be effective during the treatment period, they do not induce significant changes in the mandible’s final length or spatial position in growing patients [45, 46]. Consequently, although the immediate benefits may be similar to those seen in adult patients, the long-term effectiveness regarding permanent mandibular morphology is probably limited. However, this is a promising field of research.

## Tongue stabilizing device (TSD)

Tongue stabilizing devices (TSD) are preformed silicon-based appliances. TSD uses suction to keep the tongue forward, stretch upper airway soft tissues, and improve upper airway structure and function. As they are not reliant on the teeth for retention, TSD has been proposed as an option for patients with a reduced number or absence of teeth (hypodontia, edentulism) or compromised dental health (periodontal disease).

Deane et al., published a crossover randomized clinical trial comparing the efficacy of MAA and TSD [47]. A reduction in the AHI was observed in 91% of patients using an MAA and in 77% of patients using a TSD. This study demonstrates that a 4-week treatment period with either MAA or TSD can enhance OSA parameters, including daytime and nocturnal symptoms. The results indicate comparable efficacy of the two devices regarding AHI reduction. However, MAA showed a higher complete response rate, better overall acceptance, and greater compliance, suggesting it may be the superior treatment option for OSA in clinical practice.

Nonetheless, TSD remains a viable alternative for patients who cannot tolerate MAA or for whom MAA is inappropriate due to insufficient teeth, ongoing dental or orthodontic treatment, or a strong gag reflex. Further research is necessary to fully evaluate the role of TSD in the management of OSA.

Additionally, it was noted that most patients using TSD experienced transient tongue numbness in the morning, which typically resolved within a few hours. Some patients reported tongue tie irritation, which can be mitigated by adjusting the depth of the tongue tie cut [47]. 

## Rapid maxillary expansion

OSA in adults was first described in its modern form in 1973 [48] and was identified in children shortly thereafter [49]. It was quickly recognized that adenotonsillectomy, when indicated, had a significant positive impact on pediatric OSA [50, 51]. Adenotonsillectomy surgery is a major treatment option for OSA and was initially considered as potentially curative surgery in children [52]. Adenotonsillectomy has bee otolaryngologist n shown to improve sleep outcomes in children with sleep-disordered breathing (SDB) compared to no surgery [53]. Additionally, it has been associated with a reduction in systolic and diastolic blood pressure percentile levels, as well as a decreased likelihood of the AHI progressing to more than 3 events per hour [54], among other benefits. However, not all children are candidates for surgical intervention. Consequently, clinicians and researchers were expected to turn their attention to rapid maxillary expansion (RME).

The potential benefits of RME for patients with SDB were first proposed in a seminal article by Andrew Haas in 1970 [55], where he mentioned the indication for expansion in “cases of inadequate nasal breathing.” Yet, many years elapsed before this hypothesis was initially assessed [56]. Today, numerous unanswered questions remain interspersed among the evidence regarding the efficacy of this therapy, particularly concerning which phenotypes benefit from the procedure and which do not.

Rapid maxillary expansion significantly increases the volume of the nasal airway and oropharyngeal space [57, 58] and minimal nasal cross-sectional area [59]. This volumetric expansion tends to lead to alterations in dynamic airway parameters, including nasal airflow and resistance [60], , impacting the air passage through the airway. Despite several studies converging towards positive effects on breathing when RME is performed on OSA patients, thus rejecting the null hypothesis of no effect, some studies found no impact on breathing. This probably reflects that not all OSA patients benefit from rapid maxillary expansion [61]. 

For example, a study examining its effects on the airway found that while oropharyngeal volume increased post-treatment, these changes did not correlate with improvements in polysomnography parameters in children, such as oxygen saturation or the AHI [62]. In contrast, the same research group reported favorable neurological impacts in a preliminary study on OSA pediatric patients who underwent rapid maxillary expansion [63]. 

On the other hand, positive effects have been observed in both children [64] and adults. Specifically, children with transverse maxillary deficiency and sleep-disordered breathing post-adenotonsillectomy, including those with primary snoring and OSA, have experienced improvements in quality of life [65]. This demonstrates the potential benefit of rapid maxillary expansion in treating children with persistent snoring and transverse maxillary deficiency. These results are consistent with a previous study that aimed to determine the primary intervention for children with obstructive sleep apnea who present both a narrow maxilla and moderately enlarged tonsils. The study explored whether treatment should be initiated by an otolaryngologist or an orthodontist. It found that combining both therapies was beneficial for nearly all patients, and the sequence in which the treatments were administered had no impact on reducing the apnea-hypopnea index [66]. 

Similar and positive results in children with OSA undergoing rapid maxillary expansion have captured various nuances of potential improvements. For example, in a small sample of children with OSA, rapid maxillary expansion treatment led to significant sleep improvements after one year, including longer sleep duration, fewer stage shifts, and a reduced AHI. Patients treated with RME showed near-normal sleep architecture and improved breathing disturbances. However, their sleep microstructure and respiratory parameters did not fully recover [67]. In other words, there was an overall improvement but not a normalization of the sleep pattern.

RME not only showed immediate positive outcomes in patients with obstructive sleep apnea (OSA) and maxillary narrowing, but these outcomes also remained positive 12 years post-treatment. In a study assessing the long-term efficacy of RME in children with OSA and isolated maxillary narrowing, over an average follow-up of 12 years [64], 23 out of 31 children underwent annual evaluations, including orthodontic checks, otolaryngological exams, and repeat polysomnography (PSG), with the final assessment also involving CT imaging. These evaluations demonstrated that the outcomes of RME treatment remained stable over time, with normal clinical and PSG findings at the 12-year mark, indicating lasting positive effects. While this finding is promising, the absence of a control group necessitates a cautious interpretation of these long-term benefits.

One of the significant challenges in studies assessing the impact of rapid maxillary expansion in pediatric patients with obstructive sleep apnea (OSA) is the difficulty of conducting polysomnography in children. Consequently, many dental clinics lack the support of a hospital center capable of facilitating this examination. Additionally, when such an evaluation is conducted, not only must the child adapt to sleeping in a clinical environment, but a caregiver must also stay overnight.

Additionally, children often require desensitization visits to the clinic before the examination day. Many sleep clinics are not equipped or feel unprepared to conduct these studies on children, further limiting access to diagnosis. The future of diagnostic access and therapeutic outcome analysis lies in emerging technologies that offer high sensitivity and specificity in sleep analysis while providing greater patient comfort.

In adults, the benefits of RME have also been demonstrated. However, achieving effective maxillary expansion in adults may require adjunctive procedures. These include mini-implant-assisted rapid palatal expansion (MARPE) or distraction osteogenesis maxillary expansion (DOME). DOME involves combining MARPE with osteotomies in the maxilla to weaken the facial structure, thereby facilitating or optimizing maxillary expansion, akin to surgically assisted rapid maxillary expansion. In a selected cohort of adult OSA patients with a narrow maxilla and nasal floor, DOME [68] reduced the severity of OSA, refractory nasal obstruction, and daytime sleepiness and increased the percentage of REM sleep. A similar effect was noted in adults undergoing MARPE alone, without any osteotomies [69]. 

The ongoing debate regarding the efficacy of maxillary expansions in children may stem from numerous studies that have concentrated on secondary indicators, such as airway space, nasal airflow, resistance, and other variables, rather than on polysomnography data. In essence, numerous studies have concentrated on these covariates rather than on primary variables related to obstructive sleep apnea.

A critical aspect that requires further exploration in this field is defining what constitutes a narrow maxilla. While a posterior crossbite is a definitive indicator of a narrow maxilla, it is possible to have a narrow maxilla even without crossbites. However, there is a lack of reference parameters to delineate what is considered narrow. There is a pressing need for research that establishes normative functional and aesthetic values.

Furthermore, additional studies are required to determine how the degree of maxillary expansion influences its effects on breathing. It is hypothesized that a greater expansion may result in a more significant positive effect on sleep-disordered breathing. However, there is a notable gap in the literature, as no study has determined the relationship between the degree of expansion and its impact on breathing.

Skeletal transverse deficiencies in both maxilla and mandible could be presented, especially in those patients with syndromes and severe dentofacial deformity. Occlusion is one of the considerations on the extent of maxillary expansion. With the narrow mandible, the extent of maxillary expansion would be limited. Mandibular symphyseal distraction osteogenesis could be performed to widen the mandibular transverse dimension to relieve severe crowding and maximize the extent and efficacy of maxillary expansion [70]. 

As part of routine dental examinations, dentists can identify a small upper airway, along with other anatomical risk factors and signs of OSA [71], including the degree of palatine tonsil hypertrophy. If abnormalities are detected, the patient can be referred to an otolaryngologist for further evaluation. This screening can be easily conducted in a dental office without the need for specialized equipment. The palatine tonsils, according to Brodsky’s classification [72], are divided according to the following scale: grade I indicates that the tonsil obstructs less than 25% of the airway; grade II, 25–50%; grade III, 50–75%; and grade IV, more than 75%. Patients with more than 50% airway obstruction due to palatine tonsils (grades III and IV) are considered to have tonsillar hypertrophy. This condition is commonly associated with obstructive sleep apnea (OSA), as hypertrophy of the palatine and pharyngeal tonsils is linked to the presence of OSA [52, 73]. 

The incidence of palatine tonsil hypertrophy in children aged 4 to 17 years is 3.4%, with the peak frequency occurring between the ages of 4 and 8. However, in children aged 6 to 13 years, the incidence increases to 11%. While there is a significant association between tonsil size and snoring, tonsil size does not correlate with the severity of OSA or the success rate of surgical treatment. This suggests that other factors, such as neuromuscular components or skeletal parameters, are likely involved [74, 75]. 

On the other hand, hypertrophy of the nasal turbinates significantly influences sleep-related breathing disorders. It can lead to mouth breathing and is associated with craniofacial morphometric changes, causal factors of OSA. Therefore, a comprehensive analysis of the entire upper airway is necessary. This includes examining the nasal cavities for obstructive factors such as nasal septum deviations, hypertrophies of nasal turbinates, rhinopathies, hypertrophies of pharyngeal tonsils, palatine tonsils, and lingual tonsils, as well as assessing maxillary transverse deficiency and facial profile.

The treatment of OSA requires a multidisciplinary approach involving various healthcare professionals, including orthodontists, to effectively manage the complexity of this disorder. There is a notable gap in research directly comparing various orthodontic strategies for OSA management. More studies are needed to establish standardized protocols and explore orthodontic interventions’ long-term efficacy in treating OSA.

## Conclusion

Orthodontists play a crucial role in the management of OSA, mainly through interventions such as:

- Surgical-orthodontic Maxillomandibular Advancement is an effective treatment for OSA, especially in patients with skeletal disharmonies. It increases airway volume and reduces collapsibility but requires careful patient selection and precise phenotyping for optimal outcomes.
- Mandibular Advancement Appliances are a viable non-surgical treatment option for OSA. They improve airway patency by advancing the mandible and are associated with high patient compliance and significant symptom improvement.
- Rapid Maxillary Expansion has shown positive outcomes in children and adults with OSA and maxillary narrowing. It increases nasal and oropharyngeal airway volumes, although its efficacy can vary, and further research is needed to identify the endotypes and phenotypes that respond best to this treatment.

## Data Availability

No datasets were generated or analysed during the current study.
